# INTERPRETING ASSOCIATIONS OF SLEEP/CIRCADIAN RHYTHMS WITH COGNITIVE OUTCOMES: CHALLENGES AND FUTURE DIRECTIONS

**DOI:** 10.1093/geroni/igae098.0850

**Published:** 2024-12-31

**Authors:** Katie Stone

**Affiliations:** California Pacific Medical Center, San Francisco, California, United States

## Abstract

We and others have shown that a variety of sleep characteristics, both objective and subjective, predict cognitive decline and incident dementia. For example, degree of sleep fragmentation, circadian rest-activity rhythms reflecting timing and regularity of sleep, and sleep duration have all been identified as potential risk factors for cognitive decline and incident dementia in older adults. In addition, sleep disorders such as obstructive sleep apnea and insomnia are potential risk factors. However, several of these sleep exposures are substantially correlated, whereas others may contribute to risk of dementia via very distinct mechanisms. A more comprehensive assessment of sleep health is critical to determine which factors are most strongly linked to cognitive outcomes. Furthermore, little is known of potential risk stratification based on sex/gender and other factors. This information is critical to inform potential intervention approaches. In this presentation, a brief synthesis of the epidemiology and other evidence linking sleep and cognitive outcomes will be provided. Future directions will then be discussed, including the need for novel methods to analyze multidimensional sleep health data, exploration of potential mechanisms, larger studies to facilitate risk stratification, and ultimately, implementation of randomized trials to test whether interventions targeting specific sleep disorders and characteristics can effectively slow cognitive decline and prevent onset of dementia and Alzheimer’s Disease. We will illustrate potential intervention approaches by describing a newly implemented randomized trial, the ESSENTIAL trial (#NCT05988385), which aims to determine effects of treatment for obstructive sleep apnea on biomarkers of Alzheimer’s Disease.

